# Environmental perception and epigenetic memory: mechanistic insight through *FLC*

**DOI:** 10.1111/tpj.12869

**Published:** 2015-05-29

**Authors:** Scott Berry, Caroline Dean

**Affiliations:** 1 John Innes Centre, Norwich Research ParkNorwich, NR4 7UH, UK

**Keywords:** *Arabidopsis thaliana*, vernalization, chromatin, bistability, *FLOWERING LOCUS C*, Polycomb, non-coding RNA

## Abstract

Chromatin plays a central role in orchestrating gene regulation at the transcriptional level. However, our understanding of how chromatin states are altered in response to environmental and developmental cues, and then maintained epigenetically over many cell divisions, remains poor. The floral repressor gene *FLOWERING LOCUS C* (*FLC*) in *Arabidopsis thaliana* is a useful system to address these questions. *FLC* is transcriptionally repressed during exposure to cold temperatures, allowing studies of how environmental conditions alter expression states at the chromatin level. *FLC* repression is also epigenetically maintained during subsequent development in warm conditions, so that exposure to cold may be remembered. This memory depends on molecular complexes that are highly conserved among eukaryotes, making *FLC* not only interesting as a paradigm for understanding biological decision-making in plants, but also an important system for elucidating chromatin-based gene regulation more generally. In this review, we summarize our understanding of how cold temperature induces a switch in the *FLC* chromatin state, and how this state is epigenetically remembered. We also discuss how the epigenetic state of *FLC* is reprogrammed in the seed to ensure a requirement for cold exposure in the next generation.

**Significance Statement:**

*FLOWERING LOCUS C* (*FLC*) regulation provides a paradigm for understanding how chromatin can be modulated to determine gene expression in a developmental context. This review describes our current mechanistic understanding of how *FLC* expression is genetically specified and epigenetically regulated throughout the plant life cycle, and how this determines plant life-history strategy.

## Introduction

**Figure 1:**
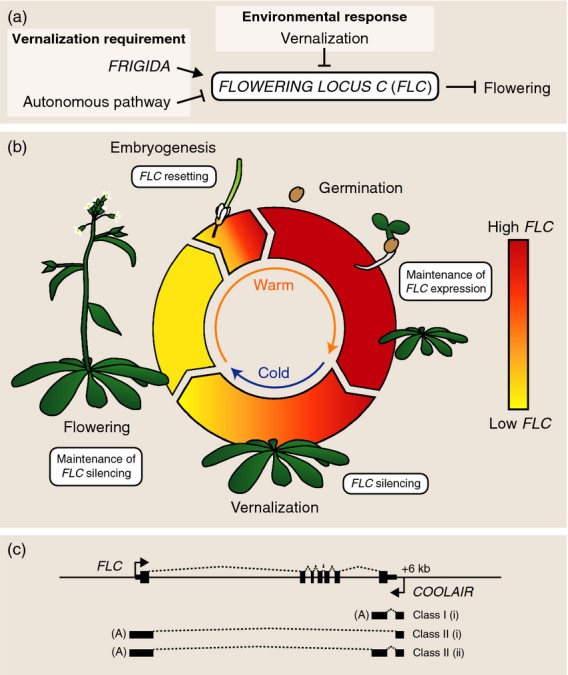
*FLC* regulation through development.(a) Genetic and epigenetic control of the floral repressor gene *FLC*.(b) Expression of *FLC* throughout the life cycle of winter-annual *Arabidopsis thaliana*. The periods of growth before and after cold exposure are periods of stable *FLC* expression, whereas vernalization and embryogenesis down-regulate and up-regulate *FLC* expression, respectively.(c) Diagram of the *FLC* genomic DNA showing sense *FLC* and antisense *COOLAIR* transcripts. Black boxes indicate exons, and dashed lines represent the splicing patterns.

Many organisms align their behaviour, metabolism and development to specific external cues. Temperature is a major environmental cue, but how this is perceived is not well understood. Plants use continuous monitoring of long-term temperature signals to infer seasonal progression in order to align development with external conditions. Unlike some environmental signals (e.g. photoperiod), temperature signals are noisy. To be capable of inferring seasonal information, plants must have systems that are capable of averaging fluctuating temperature, and also ‘remembering’ previous temperature exposure. Our understanding of how seasonal changes in temperature influence plant development is most advanced for the process of vernalization: the acceleration of flowering through exposure to prolonged cold. A requirement for vernalization ensures that plants over-winter vegetatively and flower in the following spring. Central to this process in *Arabidopsis thaliana* is regulation of the floral repressor locus *FLOWERING LOCUS C* (*FLC*) (Michaels and Amasino, 1999; Sheldon *et al*., 1999). In winter-annual Arabidopsis accessions, *FLC* is initially highly expressed and prevents transition to reproductive development before winter. *FLC* expression is repressed by prolonged cold exposure, and this repression is then epigenetically maintained until embryo development after flowering (Figure1a) (Michaels and Amasino, 1999; Sheldon *et al*., 2000). *FLC* regulation therefore provides an excellent system by which to dissect the molecular mechanisms behind temperature perception, as well as epigenetic memory and reprogramming.

In this review, we describe the key regulators of *FLC* and summarize the current understanding of *FLC* chromatin regulation at various stages of the vernalization process, including how *FLC* is repressed in response to cold exposure (cell-autonomous switching), how this cold exposure is maintained during subsequent growth in warm conditions (epigenetic memory), and how *FLC* is reprogrammed during embryo development, with opposing functions of the autonomous and FRIGIDA pathways setting the *FLC* expression level and determining reproductive strategy.

## Cell-autonomous Switching Underlies Quantitative Silencing During Cold Exposure

**Figure 2:**
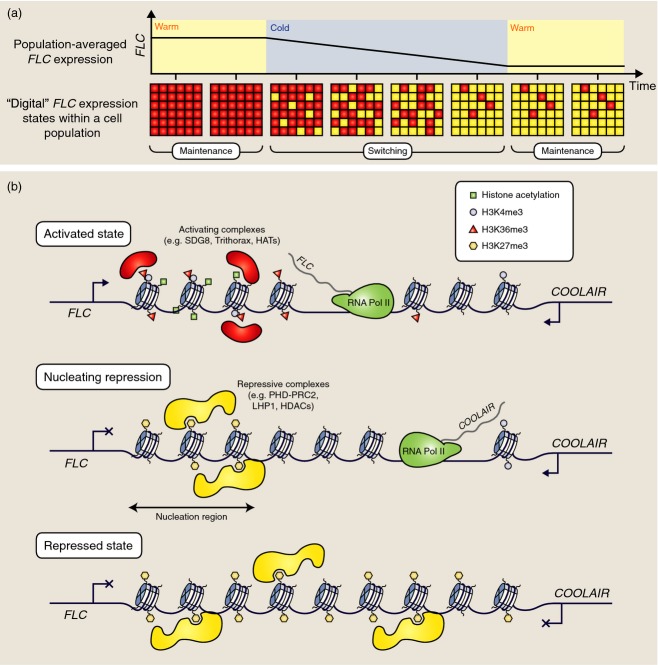
*FLC* expression and chromatin during vernalization.(a) *FLC* expression is gradually repressed during vernalization at the tissue or whole-plant level. At the cellular level, this corresponds to a gradual switching of cells from an *FLC* ‘ON’ state to an *FLC* ‘OFF’ state. *FLC* repression is stable upon return to warm conditions.(b) The high expression state of *FLC* chromatin is characterized by H3K4me3, H3K36me3, histone acetylation, and active transcription by polymerase II. During cold exposure, repression may be ‘nucleated’ by a PHD–PRC2 complex, which mediates a switch from H3K36me3-rich to H3K27me3-rich chromatin. At the same time, expression of *COOLAIR* is increased. For loci in the repressed state after cold exposure, H3K27me3 and PHD–PRC2 spread to cover the entire locus. In this repressed state, both *FLC* and *COOLAIR* transcription are reduced.

A key feature of vernalization is its quantitative nature: flowering is progressively accelerated as plants are subjected to increasing cold exposure (weeks and months). This was elegantly explained when *FLC* expression was shown to progressively decrease with increasing weeks of cold exposure (Michaels and Amasino, 1999; Sheldon *et al*., 1999). This repression is then stably epigenetically maintained after plants are returned to warm conditions. Many studies in other systems have revealed that epigenetic gene regulation systems commonly have two expression states: ‘ON’ or ‘OFF’ (Ptashne, 2004; Dodd *et al*., 2007; Veening *et al*., 2008; Ferrell, 2012). Such a gene regulation system is said to be bistable: both states are self-perpetuating under the same external conditions (Ferrell, 2002). This is also true of *FLC*: after cold exposure, *FLC* expression is actually ON or OFF in individual cells (Angel *et al*., 2011; Berry *et al*., 2015). Rather than inducing a graded reduction of *FLC* expression in each cell, cold exposure instead increases the number of cells that have switched from an ON state to an OFF state (Figure2a). When measured at the level of a tissue or whole plant, *FLC* expression appears quantitatively graded because of the large number of cells in the sample. The slow switching of cells from *FLC*-ON to *FLC*-OFF during cold exposure has been referred to as ‘digital repression’ by analogy with digital computers, which handle data as discrete ‘bits’ of information (0 or 1).

The decision to flower therefore seems to be distributed across many cells of the plant, with each cell responding independently (Angel *et al*., 2011). If all cells responded to cold exposure with an analogue (graded) *FLC* expression change, each cell would have to ‘remember’ a quantitative *FLC* expression level and pass this on through mitosis to ensure stability of epigenetic repression. Digital repression is an elegant mechanism by which plants may respond quantitatively to cold exposure without the need for individual cells to store complex quantitative information.

Cell-autonomous *FLC* repression may be converted back to an analogue flowering-induction signal at the level of a whole plant by floral integrators regulated by *FLC*. For example, one of the genes directly repressed by FLC is *FLOWERING LOCUS T* (*FT*), which is expressed in the phloem companion cells in the vasculature, and subsequently moves from the leaves to the shoot apex to induce flowering (Wigge, 2011). Movement of FT throughout the plant may act to average expression between different parts of the plant and thereby provide an indicator of ‘readiness to flower’ at the whole-plant level.

### *FLC* chromatin during the switching process

Genetic screens have been fruitful in identifying factors required for *FLC* activation and repression. Many of these protein factors act directly at the *FLC* locus to modulate the local chromatin environment in order to either promote or repress *FLC* transcription (Crevillen and Dean, 2010). Although difficult to prove conclusively, it is widely believed that post-translational modifications of histones play important roles in maintenance of both active and repressed *FLC* expression states. This hypothesis comes from two main lines of evidence. The first line of evidence is correlation: tri-methylation of histone H3 at lysine 4 and lysine 36 (H3K4me3/H3K36me3) as well as histone acetylation and histone H2B ubiquitination (H2Bub1) are commonly associated with actively transcribed genes in species from yeast to mammals (Li *et al*., 2007). These histone marks are enriched on activated *FLC* loci (Figure2b) (Yang *et al*., 2014). When *FLC* is repressed, these marks are replaced by tri-methylation at lysine 27 of histone H3 (H3K27me3) (Bastow *et al*., 2004; Sung and Amasino, 2004; De Lucia *et al*., 2008; Angel *et al*., 2011), which is a hallmark of repressed genes (Margueron and Reinberg, 2011) (Figure2b). These observations indicate that *FLC* repression involves switching chromatin from an activated state (H3K4me3/H3K36me3/H2Bub1) to a repressed state (H3K27me3). The second line of evidence is genetic: Proteins responsible for placing activating histone marks such as H3K4me3 (ATX1 and SDG25) (He *et al*., 2004; Pien *et al*., 2008; Tamada *et al*., 2009; Shafiq *et al*., 2014) or H3K36me3 (EFS/SDG8) (Kim *et al*., 2005; Zhao *et al*., 2005; Shafiq *et al*., 2014; Yang *et al*., 2014) are required for generating activated *FLC* chromatin and high levels of *FLC* expression (Figure2b). These proteins are homologous to the conserved Trithorax group of proteins required for maintenance of epigenetic active states in higher eukaryotes such as flies, nematodes and mammals (Steffen and Ringrose, 2014). For the repressive mark H3K27me3, genetic screens for components defective in maintenance of the repressed *FLC* state after vernalization led to isolation of a protein complex that is responsible for delivering H3K27me3 to *FLC* chromatin (Gendall *et al*., 2001; Sung and Amasino, 2004; Wood *et al*., 2006; Greb *et al*., 2007; De Lucia *et al*., 2008). Part of this complex is homologous to Polycomb repressive complex 2 (PRC2), which is also structurally and functionally conserved in higher eukaryotes. Core PRC2 components are estimated to be involved in maintenance of H3K27me3 at approximately 4000 genes in Arabidopsis (Zhang *et al*., 2007a; Deng *et al*., 2013). The specific PRC2 complex associated at *FLC* also includes components of the plant homeodomain (PHD) family (Sung and Amasino, 2004; Sung *et al*., 2006b; Greb *et al*., 2007; De Lucia *et al*., 2008). This PHD–PRC2 complex is physically located at *FLC* after vernalization, and is essential for maintenance of the repressed state after vernalization (Gendall *et al*., 2001; Greb *et al*., 2007) (Figure2b). Thus, chromatin-based regulation of *FLC* via the coordinated switch of histone modifications from H3K4me3/H3K36me3/H2Bub1 to H3K27me3 has emerged as a key concept underlying the epigenetic activated and repressed *FLC* expression states.

The high degree of conservation of these histone modifications (and the protein complexes that deposit them) among eukaryotes suggests that they play important conserved roles in gene regulation. However, it has been difficult to prove conclusively that particular histone modifications are absolutely required for mediating the activating or repressive effects of Trithorax or Polycomb. The main reason for this is that model organisms with a functional Trithorax/Polycomb system also have many copies of histone H3 genes. Thus, it is difficult create mutations to confirm that specific histone residues are the relevant physiological substrates of these complexes. Progress was made relatively recently through a set of experiments in *Drosophila*, in which the 23 copies of histone H3 were replaced with 12 copies of histone H3 with either wild-type lysine 27 or a mutant histone in which lysine 27 was replaced with arginine (H3-K27R) (Pengelly *et al*., 2013). Cells that only expressed H3-K27R histones and not wild-type histones failed to repress Polycomb target genes, demonstrating that PRC2 acts through histone modifications to maintain transcriptional repression of its targets.

Polycomb complexes in Arabidopsis and their functional equivalents in *Drosophila*,*Caenorhabditis elegans* and mammals have been reviewed in detail elsewhere (Arabidopsis: Holec and Berger, 2012; *Drosophila*: Steffen and Ringrose, 2014; mammals: Margueron and Reinberg, 2011). The specific complex located at *FLC* that is important for the vernalization response comprises the core PRC2 components FIE, VRN2, MSI1 and SWN or CLF, as well as the PHD proteins VRN5, VIN3 and VEL1 (Sung and Amasino, 2004; Sung *et al*., 2006b; De Lucia *et al*., 2008). CLF and SWN are homologues of E(z) in *Drosophila* (EZH2 in mammals). This is the enzymatic subunit that catalyses H3K27me3 through its SET domain (Cao *et al*., 2002). FIE is homologous to Esc in *Drosophila* (EED in mammals), which has been shown to specifically recognize H3K27me3. In the context of PRC2, H3K27me3 binding by EED results in allosteric activation of PRC2 H3K27me3 methyltransferase activity (Margueron *et al*., 2009). The zinc finger protein VRN2 (Su(z)12) and the WD40-domain protein MSI1 (p55) are core complex components that make contacts with histones and enhance PRC2 catalytic activity. LHP1 is also physically located at *FLC* chromatin in the repressed state, and appears to be important in maintenance of repression (Mylne *et al*., 2006; Sung *et al*., 2006a; Turck *et al*., 2007). LHP1 is capable of binding H3K27me3 (Turck *et al*., 2007; Zhang *et al*., 2007b) and also interacts with the PRC2 subunit MSI1 (Derkacheva *et al*., 2013).

When plants are exposed to cold, *VIN3* expression is induced (Sung and Amasino, 2004), and VIN3 accumulates as part of a PHD–PRC2 complex downstream of the *FLC* transcription start site (De Lucia *et al*., 2008). This region is referred to as the nucleation region, and consists of approximately three nucleosomes centred over exon 1/the start of intron 1 (Figure2b). This complex results in coordinated loss of H3K4me3/H3K36me3 and gain of H3K27me3 at the nucleation region (Yang *et al*., 2014). In parallel with this change in chromatin state, transcriptional down-regulation of *FLC* and up-regulation of *COOLAIR* antisense transcripts occur (Swiezewski *et al*., 2009). These transcriptional changes occur independently of VIN3 (Swiezewski *et al*., 2009; Helliwell *et al*., 2015). The down-regulation of *FLC* sense transcription early during cold exposure may be an important prerequisite for recruitment of Polycomb complexes to the nucleation region. Indeed, it was shown that H3K27me3 is effectively ‘wiped out’ when transcription across *FLC* intron 1 is driven by an artificial inducible promoter in transgene experiments (Buzas *et al*., 2011). Further support for the idea that PRC2 is capable of targeting transcriptionally repressed loci ‘by default’ has come from recent experiments in mammalian embryonic stem cells (Riising *et al*., 2014). In these experiments, it was found that global transcriptional inhibition was sufficient to induce ectopic PRC2 recruitment to Polycomb target genes that were not normally silenced in embryonic stem cells (Riising *et al*., 2014). This study also showed that PRC2 was dispensable for initial transcriptional shutdown of many genes that are switched off during *in vitro* differentiation of embryonic stem cells. It appears that PRC2 may act to sample permissive chromatin sites and to silence those that are not transcriptionally active (Klose *et al*., 2013). Thus, transcription itself may form a key component of the ‘activated state’, which antagonizes Polycomb silencing. An interesting finding relevant to this proposal is that PRC2 interacts with nascent RNA at both inactive and active loci across the mammalian genome (Kaneko *et al*., 2013, 2014).

Exactly how Polycomb complexes are targeted to specific genomic locations such as the *FLC* nucleation region remains the subject of intense research. Several studies have identified sequences in the first intron as being important for vernalization (Figure1c) (Sheldon *et al*., 2002; Sung *et al*., 2006a; Angel *et al*., 2011). In *Drosophila*, the well-studied Hox loci contain specific DNA sequences that are recognized by sequence-specific DNA binding proteins. These proteins then provide a targeting platform for the Polycomb and Trithorax complexes. The DNA elements are called Polycomb response elements (Steffen and Ringrose, 2014). To date, specific sequences capable of acting as epigenetic memory elements in the same way as *Drosophila* Polycomb response elements have not been identified in plants or mammals. The discovery of long non-coding RNA and the RNA-binding ability of Polycomb complexes led to the hypothesis that long non-coding RNAs may act as ‘recruiters’ of PRC2 (Tsai *et al*., 2010). This is the proposed mechanism of action of a sense long non-coding RNA (*COLDAIR*) transcribed from *FLC* intron 1 (Heo and Sung, 2011). However, the *COLDAIR* sequence is not well conserved in *FLC* orthologues from close relatives of *A. thaliana*, such as *Arabidopsis lyrata* and *Capsella rubella* (Castaings *et al*., 2014), and PRC2 RNA binding appears to be quite promiscuous (Davidovich *et al*., 2013; Kaneko *et al*., 2013). Therefore, it is currently unclear exactly how long non-coding RNAs could provide the required specificity to recruit PRC2 to specific genomic locations.

**Figure 3:**
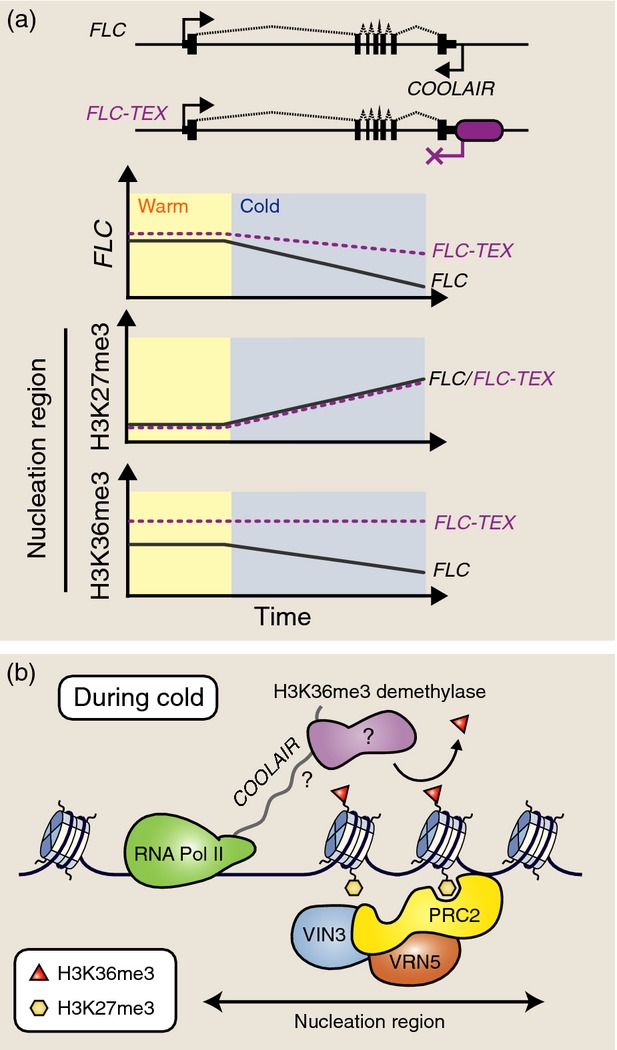
The role of *COOLAIR* in vernalization.(a) Terminator exchange (TEX) lines have the *COOLAIR* promoter replaced with the *RUBISCO* (*RBCS*) terminator. *FLC-TEX* is not transcriptionally repressed as rapidly as the control (*FLC*). While H3K27me3 accumulation at the nucleation region of *FLC* appears unaffected in *FLC-TEX*, H3K36me3 is not removed from *FLC* chromatin during cold exposure.(b) Nucleation of H3K27me3 at *FLC* during cold exposure requires a PHD–PRC2 complex containing VIN3 and VRN5. *COOLAIR* does not affect H3K27me3 nucleation, but may act to stabilize an H3K36me3 demethylase at the nucleation region.

The *COOLAIR* promoter and first exon sequences are highly conserved in perennial relatives of *A. thaliana*, as is cold induction of *COOLAIR* expression (Castaings *et al*., 2014), suggesting a potentially conserved function in vernalization. While *COOLAIR* transcripts do not appear to be absolutely required for the vernalization response (Helliwell *et al*., 2011; Csorba *et al*., 2014), recent experiments have uncovered a role for *COOLAIR* in the coordinated switch between H3K27me3 and H3K36me3 at the nucleation region (Csorba *et al*., 2014) during cold exposure. This study used a transgenic *FLC* construct in which the *COOLAIR* promoter was replaced with the terminator from the *RUBISCO* gene. These are referred to as terminator exchange or TEX lines. Transgenic plants carrying the TEX construct showed very low *COOLAIR* expression that was no longer induced during cold exposure. There were two striking effects of reducing *COOLAIR* transcription on the behaviour of *FLC* during cold exposure. First, *FLC* sense transcriptional down-regulation was not observed to take place as rapidly in TEX as in control lines with a functional *COOLAIR* promoter (Figure 3a). Even more striking, however, was the total lack of reduction in H3K36me3 during cold exposure in TEX lines (Csorba *et al*., 2014). Thus, *COOLAIR* (or the process of antisense transcription) appears to be required during cold exposure to ensure removal of activating chromatin marks and to mediate *FLC* transcriptional down-regulation. This raises the possibility of the existence of an H3K36me3 demethylase (currently hypothetical), whose targeting to *FLC* depends on antisense *COOLAIR* transcripts (Figure3b).

While long non-coding RNAs may help to localize protein complexes, they may also be involved in eviction of protein complexes. This process was recently shown to be important for regulation of the chromatin state at a Polycomb response element in *Drosophila* (Herzog *et al*., 2014). In this case, both sense and antisense non-coding RNAs bind PRC2 and inhibit its enzymatic activity *in vitro*, but only the antisense non-coding RNA binds PRC2 *in vivo*. Furthermore, specific over-expression of the reverse strand was sufficient to evict PRC2 from chromatin and activate the Polycomb response element (Herzog *et al*., 2014). The role of non-coding transcription in evicting chromatin modifiers has also been proposed to help define heterochromatin boundaries in the yeast *Schizosaccharomyces pombe* (Keller *et al*., 2013). It is therefore possible that *COOLAIR* transcription during cold exposure functions to remove a complex required for H3K36me3 addition at the nucleation region.

### ‘Digital’ nucleation enables buffering of noisy temperature signals

Mathematical modelling of *FLC* chromatin dynamics before, during and after vernalization has suggested a key role for the H3K27me3 nucleation peak in ‘pushing’ the state of the locus from the activated to the repressed state (Angel *et al*., 2011). Nucleation of repression appears to involve a switch from H3K36me3 to H3K27me3, as these have opposing profiles in the *FLC* nucleation region (Yang *et al*., 2014). Other experiments also support mutual exclusion of H3K36me3 and H3K27me3: they rarely co-exist on the same histone tail (Johnson *et al*., 2004; Voigt *et al*., 2012; Yang *et al*., 2014), the antagonism is functionally important (Yuan *et al*., 2011; Yang *et al*., 2014), and lack of H3K36me3 results in a fully silenced state at *FLC* even in the absence of cold exposure (Yang *et al*., 2014). However, the absence of an absolute mirror profile between H3K27me3 and H3K36me3 across the whole *FLC* locus, predicted from modelling, suggests their antagonistic roles are a necessary but not sufficient component of the mechanism enabling switching between, and inheritance of, epigenetic states (Yang *et al*., 2014). It is currently unknown whether H3K27me3 at the nucleation region during cold exposure occurs in all cells equally (analogue) or in an all-or-nothing fashion (digital), i.e. does H3K27me3 at the nucleation region increase gradually at all *FLC* loci at similar rates, or does the proportion of cells that have a strong, persistent H3K27me3 nucleation peak increase gradually during cold exposure?

These two possibilities have recently been considered using mathematical modelling of *FLC* chromatin (Angel *et al*., 2015). In an analogue nucleation model, the probability of switching a locus from activated to repressed depends on the height of the nucleation peak, which increases during cold exposure at approximately the same rate in all cells. In the digital nucleation model, cells either have a nucleation peak or do not have a nucleation peak, with the fraction of ‘nucleated’ cells increasing for longer cold exposures. In the digital model, only cells with a nucleation peak make the switch to the silenced state (with high probability) after cold exposure. This study showed that *FLC* silencing with analogue nucleation is not sensitive to short periods of cold exposure because the small H3K27me3 peak generated in all cells is not sufficient to switch the overall chromatin state of the locus. Conversely, if temperature is registered using a digital nucleation peak, the peak can easily ‘flip’ the state of the gene after any length of cold exposure because the peak ‘height’ is the same in any nucleated cell, regardless of the duration of cold exposure (Angel *et al*., 2015). The finding that analogue temperature registration does not perform well for short cold exposures has strong implications for how plants perceive cold in fluctuating temperatures. Whereas digital temperature registration may function to switch states at a certain probability when plants are exposed to cold, analogue temperature registration requires longer periods of cold exposure to generate effective H3K27me3 peaks in all cells. It therefore follows that a digital nucleation mechanism is much better at buffering fluctuating temperature regimes such as those normally experienced in natural environments. The authors tested the response of *FLC* expression to a fluctuating temperature regime, with 4-day breaks between short cold spells. They found that plants respond similarly to interrupted and non-interrupted cold exposure. This strongly supports the hypothesis that plants register temperature signals in a digital manner, with all-or-nothing H3K27me3 peaks at the *FLC* nucleation region arising during cold exposure.

An experimental observation in support of digital nucleation is the physical clustering of *FLC* loci that occurs during cold exposure (Rosa *et al*., 2013). Live-cell imaging in an *FLC–lacO*/*lacI* system was used to monitor changes in the physical position of *FLC* loci within the nucleus during vernalization. *FLC–lacO* alleles were found to physically cluster during cold exposure, and generally remain clustered after plants are returned to warm conditions (Rosa *et al*., 2013). Clustering depends on the presence of PHD–PRC2 components necessary for switching *FLC* to the silenced state, but not on LHP1. The quantitative increase in clustering with cold exposure paralleled the quantitative increase in H3K27me3 at the nucleation site, suggesting a tight connection between the switching mechanism and changes in nuclear organization.

How fluctuating temperatures are translated into digital silencing is an important question that is currently being addressed. Temperature registration in a field environment was studied in a two-year census of natural populations of the perennial plant *Arabidopsis halleri* (Aikawa *et al*., 2010). Expression of flowering time genes was measured every week in plants growing in natural field conditions. *FLC* expression was found to decrease gradually as the winter progressed. The authors attempted to correlate *FLC* expression with the fluctuating temperature regime experienced by the plants, and found that *FLC* expression levels were best explained by the cumulative mean daily temperature over the preceding 6 weeks.

## Epigenetic Maintenance of the Silenced State

The involvement of histone modifications in regulating expression of Polycomb target genes such as *FLC* has given rise to the hypothesis that histone modifications are not only important mechanistically for achieving transcriptional repression but may also be carriers of epigenetic memory (Kaufman and Rando, 2010; Moazed, 2011; Steffen and Ringrose, 2014). The idea is that once *FLC* chromatin is covered in histone modifications such as H3K27me3, these modifications are sufficient to recruit the machinery (such as PRC2) to ensure that they are maintained indefinitely at that locus despite the noisy processes of nucleosome turnover and H3K27me3 demethylation. The concept of histone modifications as carriers of epigenetic information has become so embedded in current thinking that histone modifications are commonly referred to as ‘epigenetic marks’, implying that a region of such marks has the intrinsic capacity to instruct its own maintenance and inheritance in daughter cells. The debate over whether histone modifications are the cause or consequence of epigenetically heritable transcriptional states is on-going (Ptashne, 2007; Kaufman and Rando, 2010; Henikoff and Shilatifard, 2011).

While histone modifications may act as carriers of epigenetic memory at some loci, it is worth considering other possibilities for epigenetic gene regulation that are not dependent on histone modifications. We discuss two main classes of memory mechanisms: cis memory and trans memory (Bonasio *et al*., 2010). In cis memory, epigenetic information is physically located at chromatin, possibly in the form of DNA methylation or histone modifications. In trans memory, epigenetic information is stored in the concentration of a diffusible factor, such as a transcriptional repressor.

### Trans memory

**Figure 4:**
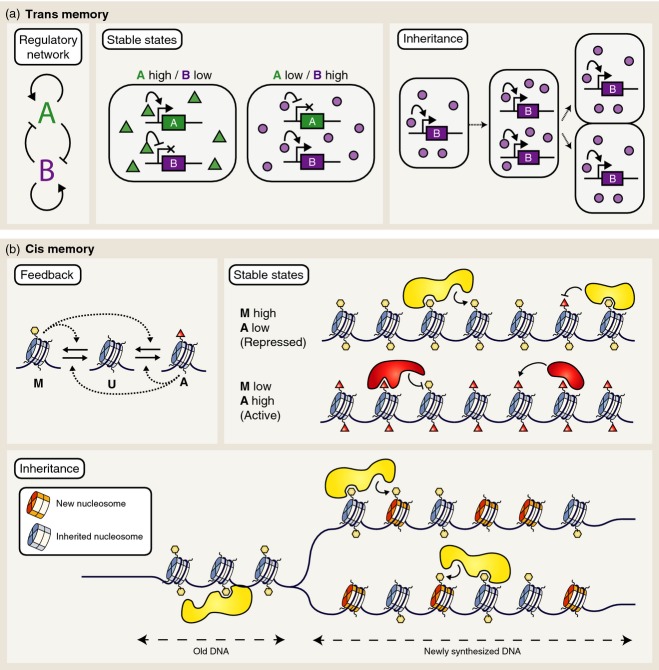
Mechanisms of epigenetic memory.(a) Trans memory. A hypothetical gene regulatory network containing transcription factors A and B is shown. These mutually repress each other’s transcription. Stable states of this network are encoded by the global concentrations of these factors. Inheritance in trans memory occurs by passing on high concentrations of one of the diffusible factors to the daughter cells.(b) Cis memory. A hypothetical three-state model of modified histones is shown. Each of the ‘A’ and ‘M’ marks may recruit more modifications of the same type to nearby nucleosomes. Stable states are encoded by the local proportion of ‘M’ and ‘A’ histone marks. Inheritance in cis memory occurs by passing on nucleosomes to daughter DNA strands at the replication fork. Newly incorporated (unmodified) nucleosomes may be modified in the same way as the parental DNA because the inherited modifications recruit the relevant modification complexes.

In principle, both cis and trans memory mechanisms are capable of generating heritable bistable gene expression states. Trans memory is commonly used in bacterial systems such as lambda phage (Oppenheim *et al*., 2005), the *Escherichia coli lac* operon (Vilar *et al*., 2003) and for bet-hedging in bacterial populations (Veening *et al*., 2008). Trans memory uses trans-acting feedback loops to generate multiple stable expression states. A simplified trans memory network is shown in Figure4(a). The system comprises two genes, A and B, that mutually repress each other’s transcription and also auto-activate. For simplicity, A and B may be thought of as transcription factors. If gene A is expressed highly, then gene B will be repressed, and vice versa, which leads to two (mutually exclusive) stable states (A high/B low or A low/B high) (Figure4a). Furthermore, it is easy to see how such a trans memory system leads to inheritance of expression states in daughter cells. As the DNA is replicated in a ‘low A/high B’ cell, protein B continues to activate its own expression, maintaining a constant concentration as the cell grows. When the cell divides, molecules of B are divided roughly equally between daughter cells, where they continue to activate expression of gene B (and repress expression of gene A). Thus, the low A/high B state is inherited.

While this is an artificially simple example, a complex gene regulatory network with many components and feedbacks may also generate epigenetically stable expression states by means of trans-regulation. Reference to sequence-specific DNA-binding proteins such as transcription factors is convenient for explaining the concept of trans memory; however, other more exotic gene regulation mechanisms such as those involving trans-acting small RNAs also function in conceptually similar ways (Stuwe *et al*., 2014).

### Cis memory

Like trans memory, cis memory must be bistable, i.e. it must have two self-perpetuating alternative gene expression states. However, the key difference compared with trans memory is that the information carriers are physically located at the gene itself. DNA methylation is a more well-established carrier of epigenetic memory *in cis* than histone modifications (Chan *et al*., 2005). However, DNA methylation does not appear to be involved in *FLC* regulation during vernalization (Finnegan *et al*., 2005). Whether histone modifications can act as heritable elements that ensure propagation of activated and repressed states is still an open question. A pioneering theoretical model based on this hypothesis was initially developed to describe epigenetic memory at the silent mating-type region of the yeast *Schizosaccharomyces pombe* (Dodd *et al*., 2007). A conceptually similar model was later applied to the study of *FLC*, and demonstrated quantitative agreement with experimental chromatin immunoprecipitation data regarding switching of bistable epigenetic states at *FLC* through vernalization (Angel *et al*., 2011).

The model postulates that nucleosomes exist in one of three states: M (methylated/repressive), U (unmodified) and A (activating) (Dodd *et al*., 2007). Like trans memory, bistability in the cis memory model requires feedback. The feedback is implemented in the model in the following manner: modified nucleosomes such as M (e.g. H3K27me3) have the ability to recruit protein complexes (such as PRC2) to similarly modify nearby nucleosomes. This positive feedback of histone modifications tends to cause the region of chromatin to be predominantly covered in either M or A nucleosomes. The model therefore generates two stable chromatin ‘states’. The underlying molecular explanation for this ‘M recruits more M’ feedback mechanism in the case of PRC2 is thought to be that PRC2 contains one subunit that binds to H3K27me3 and another subunit that adds H3K27me3 (Hansen *et al*., 2008; Margueron *et al*., 2009). The molecular basis of the other feedbacks in the model is less well understood, but nonetheless the theoretical requirement for these feedbacks suggests interesting directions for future experiments.

Why does the model need so many feedbacks? The key problem with storing epigenetic information in histone modifications is that the nucleosomes may be removed and replaced over time scales of hours (Jamai *et al*., 2007; Deal *et al*., 2010). If the marks are not re-written on a shorter time scale than this, chromatin states are not maintained, even within a cell cycle. The second major hurdle that a model of histone modification-based memory must overcome is inheritance through cell division. How is it that this model ensures inheritance of the ‘high M’ and ‘high A’ chromatin states? It is well known from many experiments (mainly in yeast and *Drosophila*) that nucleosomes are inherited semi-conservatively as DNA is replicated (Annunziato, 2005), i.e. nucleosomes are shared between daughter strands (Figure4b). The hypothesis is that the histone modifications are also shared equally between daughter strands. If the spaces between the inherited nucleosomes are filled with new unmodified nucleosomes, a newly replicated DNA strand will have approximately half as many histone modifications as the original region of chromatin. The feedbacks in the model ensure that these modifications are sufficient to recruit the required protein complexes to ‘fill in the gaps’, and thus propagate the epigenetic state (Dodd *et al*., 2007) (Figure4b). While inheritance of nucleosomes during DNA replication is well established, recent experiments in *C. elegans* have shown that histone modifications (H3K27me3) may also be passed on to daughter chromosomes in the absence of PRC2 (Gaydos *et al*., 2014), supporting the hypothesis that inherited histone marks may underlie epigenetic memory. In addition, it has been proposed that Polycomb and Trithorax proteins themselves are passed on locally at the DNA replication fork (Francis *et al*., 2009; Petruk *et al*., 2012), which may further contribute to the epigenetic stability of chromatin domains in a cis memory mechanism.

### Memory is stored in cis at *FLC*

The components required for switching and maintenance of *FLC* expression states that were isolated using unbiased genetic screens suggest the existence of a chromatin-based mechanism for epigenetic memory. However, until recently, it remained difficult to exclude the existence of trans factor-based memory because FLC protein (a MADS-box transcriptional repressor) or non-coding RNA produced at the *FLC* locus could feed into a bistable trans-regulatory network. A recent study used two distinguishable fluorescent reporters of *FLC* expression in the same cells to investigate the cis-memory storage capability of *FLC* chromatin (Berry *et al*., 2015). It was shown that, after vernalization, two copies of *FLC* in the same cell may be in different expression states, i.e. one of the *FLC* reporters may be repressed in the same cell as the other reporter is active. Furthermore, the authors found that this ‘mixed’ expression state is stably inherited through several cell divisions. This indicates that the epigenetic memory of *FLC* expression is physically located in the local chromatin environment (Berry *et al*., 2015). It is therefore the chromatin state, rather than concentrations of diffusible trans factors, that dictates *FLC* transcription after vernalization. Together with previous results, this finding supports the hypothesis that histone modifications such as H3K27me3 are important components of epigenetic memory.

### Instructive and responsive chromatin

In the case of cis memory, the chromatin state is responsible for instructing its own inheritance, and may therefore be referred to as ‘instructive’. In the case of trans memory, chromatin may still play a vital role in mediating the effects of trans factor binding events to orchestrate gene regulation. However, in this case, chromatin is ‘responsive’ to trans factors rather than being the key epigenetic memory element.

*FT* is an example of Polycomb-repressed chromatin in Arabidopsis that may be ‘responsive’ rather than ‘instructive’. The *FT* locus is covered in high levels of H3K27me3 in the repressed state (Adrian *et al*., 2010), and repression depends partly on LHP1, which binds *FT* chromatin (Takada and Goto, 2003). However, the memory of repression is at least partly maintained by high concentrations of repressive trans factors, including FLC and SHORT VEGETATIVE PHASE (SVP) (Hepworth *et al*., 2002; Li *et al*., 2008).

### Natural variation in epigenetic memory

DNA sequence variation at *FLC* influences epigenetic silencing, and is likely to be very useful in elucidating chromatin switching and maintenance mechanisms (Li *et al*., 2014). Genomic sequence analysis of > 1000 accessions identified 20 *FLC* haplotypes that are defined only by non-coding polymorphisms. There were five high-frequency groups in the worldwide population. These multiple, functionally distinct *FLC* haplotypes appear to have been maintained in the population, and probably contribute to the broad geographical and niche ranges of *A. thaliana* accessions. The various groups had distinct epigenetic silencing characteristics. Two vernalized quickly, with a relatively short period of cold exposure (4–6 weeks) being sufficient for full epigenetic silencing. The remaining three vernalized slowly, with a longer period of cold exposure (10–12 weeks) required for silencing (Shindo *et al*., 2006; Li *et al*., 2014). When transgenes containing the various *FLC* haplotypes were transformed into a common genetic background, the same differences in regulation remained, demonstrating that the non-coding sequence polymorphisms influenced the rate of *FLC* epigenetic silencing (Li *et al*., 2014). Detailed analysis of which sequence polymorphisms affect epigenetic memory was performed for the Northern Swedish accession Lov-1 (Coustham *et al*., 2012). The Lov-1 accession is particularly unresponsive to short cold periods: 4 weeks is not sufficient to stimulate flowering, and seedlings need 12 weeks of cold exposure to fully saturate the vernalization requirement (Shindo *et al*., 2006; Strange *et al*., 2011). Transgenic studies analysing *FLC* constructs with different combinations of polymorphisms from the Lov-1 and Col-0 alleles showed that four distinct single nucleotide polymorphisms in the nucleation region of Lov-1 *FLC* accounted for a large proportion of the requirement for extended cold exposure (Coustham *et al*., 2012).

## Reprogramming *FLC* Expression in the Seed

As the germ line in plants arises from the somatic tissues, extensive epigenetic reprogramming occurs prior to the next generation. This includes *FLC*, whose expression needs to be ‘reset’ at some stage after the floral transition to ensure a vernalization requirement in each generation. Unlike the slow quantitative switching from the ‘ON’ to ‘OFF’ state during cold exposure, the reprogramming occurs relatively synchronously in the developing seeds. *FLC* expression increases throughout embryogenesis, and reaches a maximum when the seed has fully formed (Sheldon *et al*., 2008; Choi *et al*., 2009). The activation in the early multicellular embryo occurs independently of *FRI* and autonomous pathway genes, which have major effects from late embryogenesis onwards (Choi *et al*., 2009). While *FLC* expression is cell-autonomous after vernalization, it is currently not clear whether this is also true during resetting of *FLC* expression during embryogenesis and then during subsequent development in the next generation.

**Figure 5:**
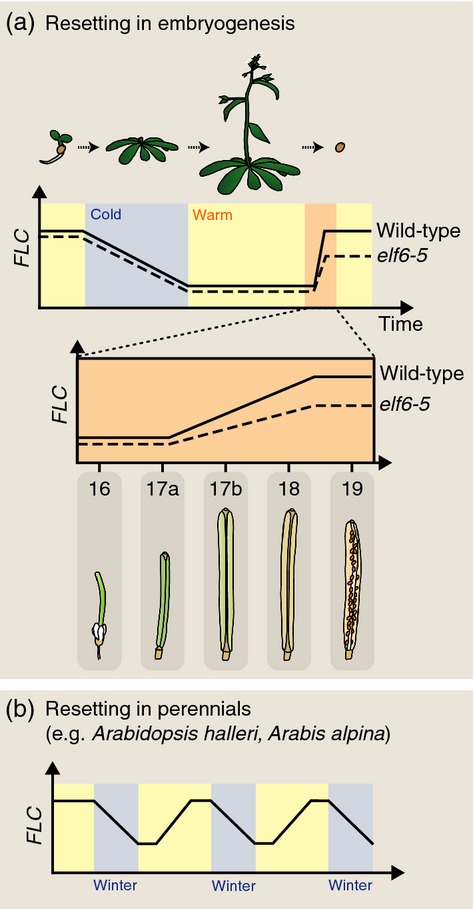
*FLC* resetting.(a) *FLC* expression is reset during embryogenesis. *FLC* expression increases from silique stage 17a (early globular embryo) until stage 19 (mature seed) (Roeder and Yanofsky, 2006). Resetting after vernalization is disrupted in the H3K27me3 demethylase mutant *elf6*-*5*.(b) Perennials such as *Arabidopsis halleri* and *Arabis alpina* show down-regulated *FLC* expression each winter; *FLC* expression is then reset in all meristems, preventing flowering in those that have not yet expressed floral activators.

A genetic screen for mutants that fail to fully reset *FLC* expression in the generation after vernalization revealed a role for *EARLY FLOWERING 6* (*ELF6*) (Crevillen *et al*., 2014) in epigenetic reprogramming (Figure5a). *FLC* expression was only slightly lower in *elf6*-*5* than the wild-type if plants were not subjected to cold exposure (provided the previous generation was not vernalized). However, the increase in *FLC* expression normally observed during resetting was much lower in *elf6*-*5*. This reduced expression and early flowering was inherited over subsequent generations, thus the *elf6*-*5* mutation caused partial trans generational inheritance of vernalization-induced *FLC* repression (Crevillen *et al*., 2014). *ELF6* encodes a jumonji domain-containing protein with H3K27me3 demethylase activity that is highly expressed in flowers and embryos (Crevillen *et al*., 2014). This suggests that the hypomorphic mutation in *elf6*-*5* leads to failure to fully remove the H3K27me3 modifications induced through vernalization. Inheritance of this repressed state in the germ line supports the role of H3K27me3 as a carrier of epigenetic information.

The timing of resetting differs in annual plants compared to perennial relatives (Wang *et al*., 2009). In perennials, only a subset of meristems switch to reproductive development at any one time. This appears to be achieved through combination of a requirement for specific environmental conditions with variation in meristem reproductive competence (Turck and Coupland, 2014). A homologue of *FLC*,*PERPETUAL FLOWERING 1* (*PEP1*), is needed in *Arabis alpina* for perennial flowering (Wang *et al*., 2009). Like *FLC* in the annual *A. thaliana*,*PEP1* expression in *Arabis alpina* decreases during cold exposure in all meristems. In reproductive-competent meristems, this is sufficient to induce expression of downstream floral activators. However, unlike *A. thaliana*, there is only transient epigenetic memory of prolonged cold exposure (Figure5b). On return to warm conditions, *PEP1* expression is reset, preventing flowering in those meristems that are not yet expressing floral activators. Mathematical modelling has suggested that the relative rates of addition/removal of activating and repressive histone modifications at *FLC*/*PEP1* after cold exposure may account for differences in stability of the repressed epigenetic states (Satake and Iwasa, 2012). The genetic determinants of earlier resetting in *Arabis alpina* compared to *A. thaliana* are yet to be fully determined, but appear to involve both cis regulatory sequence variation and differences in trans factors (Castaings *et al*., 2014).

### Opposing functions of FRIGIDA and autonomous pathways set the *FLC* expression level

**Figure 6:**
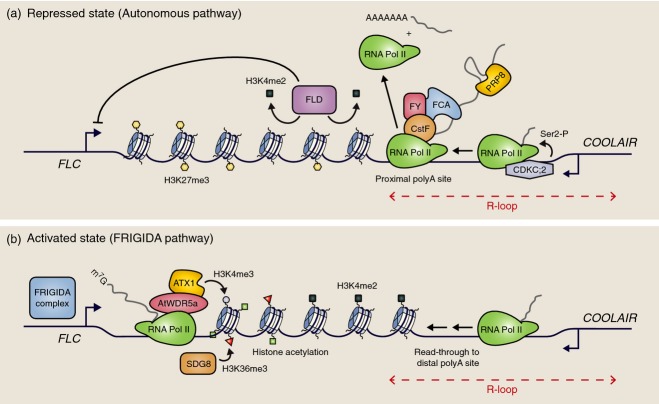
Autonomous pathway/FRIGIDA ‘tug of war’ to set and maintain *FLC* expression.(a) Autonomous pathway components FCA, FY, CstF, CDKC;2 and PRP8 lead to a repressed state of *FLC* expression. These factors promote proximal splicing and polyadenylation of *COOLAIR*, which leads to FLD recruitment, H3K4me2 demethylation and H3K27me3 methylation. and results in reduction of *FLC* transcription. An R-loop extending from the *COOLAIR* promoter to the proximal polyadenylation site represses *COOLAIR* transcription, whilst CDKC;2 promotes *COOLAIR* transcription.(b) FRIGIDA (FRI) activates *FLC* transcription, thus opposing the autonomous pathway activity. FRI activity leads to increased capping of the nascent *FLC* transcript, increased H3K4 and K36 methylation, decreased H3K27me3 and increased distal polyadenylation of *COOLAIR*.

The expression level to which *FLC* is reset in the seed has significant consequences on the reproductive strategy of the plant. High *FLC* expression leads to a strong vernalization requirement, requiring over-wintering of plants before flowering. Low *FLC* expression relaxes the requirement for vernalization, and leads to the potential for rapid cycling, thus achieving multiple generations per year. The expression level of *FLC* is set by the opposing activities of the *FRIGIDA* (*FRI*) pathway (activating) and the autonomous pathway (repressive) (Koornneef *et al*., 1998). These participate in a ‘tug-of-war’ to set and maintain the *FLC* expression from late embryogenesis and into vegetative growth (Figure6). The genetic and molecular analyses of the autonomous and FRIGIDA pathways have been reviewed previously (Crevillen and Dean, 2010; Ietswaart *et al*., 2012). Here, we focus on how opposing *FLC* chromatin states are established by the two pathways to set the transcription level of *FLC*. This occurs in the developing embryo and is maintained through vegetative development, unless cold-induced silencing occurs.

The autonomous pathway represses *FLC* expression, resulting in early flowering (Koornneef *et al*., 1991). Components that function in this pathway include the RNA-binding proteins FCA (Macknight *et al*., 1997; Quesada, 2003) and FPA (Schomburg *et al*., 2001), the 3’ processing factors FY (Simpson *et al*., 2003) and Cstf77/Cstf64 (Liu *et al*., 2010), the core spliceosome subunit PRP8 (Marquardt *et al*., 2014), and the chromatin regulators FLD (Liu *et al*., 2007), pTEFb (Wang *et al*., 2014) and LD (Lee *et al*., 1994). Low *FLC* expression is associated with a specific chromatin state: low acetylation, H3K4me2, H3K36me3 and high H3K27me3 (He *et al*., 2003; Yang *et al*., 2014) (Figure6a). Conversely, high *FLC* expression requires FRIGIDA complex components: Trithorax-like SET-domain proteins ATX1 (Pien *et al*., 2008), SDG25 (Berr *et al*., 2009; Tamada *et al*., 2009), SDG7 (Lee *et al*., 2015) and SDG8 (Yang *et al*., 2014), and the WD40-domain protein AtWDR5a (Jiang *et al*., 2009) (Crevillen and Dean, 2010). The activated chromatin state is characterized by high acetylation, low H3K27me3, high H3K4me3/H3K36me3 in the nucleation region, and accumulation of H3K4me2 in the gene body (Figure6b) (He *et al*., 2003; Liu *et al*., 2007; Yang *et al*., 2014). At elevated temperature, the H3K27 demethylase JMJ30 is also required for high *FLC* expression (Gan *et al*., 2014).

Central to the autonomous pathway function is a co-transcriptional coupling between *COOLAIR* processing and the chromatin state at *FLC* (Figure6a). *COOLAIR* is alternatively spliced and polyadenylated (Liu *et al*., 2007, 2010; Hornyik *et al*., 2010; Marquardt *et al*., 2014; Wang *et al*., 2014). Autonomous pathway components promote use of both the proximal *COOLAIR* splice acceptor site and the proximal polyadenylation site, and result in FLD-dependent H3K4me2 demethylation in the *FLC* gene body. This particular chromatin state then reinforces choice of the proximal splice site and polyadenylation site (Marquardt *et al*., 2014), possibly via a kinetic coupling mechanism (Alló *et al*., 2009). Slow transcription has been linked to proximal splice site choice and early termination (de la Mata *et al*., 2003; Hazelbaker *et al*., 2013).

High expression of *FLC* is promoted by FRIGIDA function through a Trithorax-like mechanism (Figure6b). FRIGIDA associates with components of the RNA 5’ cap-binding complex for the nascent transcript, and leads to a higher proportion of the *FLC* transcripts containing a 5’ cap. Natural polymorphisms that alter splicing of distally polyadenylated *COOLAIR* promote *FLC* transcription, also via an influence on the capping of the nascent transcript (Li *et al*., 2015). The directly opposing functions of the autonomous and FRIGIDA pathways therefore mechanistically converge on the co-transcriptional link between *COOLAIR* processing and recruitment of chromatin regulators. Natural non-coding polymorphisms that define the functionally distinct *FLC* haplotypes (Li *et al*., 2014) may alter one of these opposing pathways, with small changes in either being magnified by the opposing effect of the other.

Given the central role of *COOLAIR* in regulation of *FLC* in both warm and cold conditions, it is interesting to consider specific regulators of *COOLAIR* expression. *COOLAIR* transcription is initiated from a non-canonical promoter within a genomic region carrying termination sequences for the sense transcript, a feature that is frequently found in yeast (Murray *et al*., 2012). Small RNAs (24- and 30-mers) homologous to the *COOLAIR* promoter have been detected, and these are required for maintenance of a small patch of H3K9me2-modified chromatin just upstream of the major *COOLAIR* start site in reproductive tissues (Swiezewski *et al*., 2007). To enable genetic screens for specific *COOLAIR* regulators, the *COOLAIR* transcript was modified to contain a luciferase-coding sequence (Swiezewski *et al*., 2009). These studies identified a homeodomain protein (AtNDX1) that binds to single-stranded DNA in a non-sequence-specific manner (Sun *et al*., 2013). This homeodomain protein stabilizes an RNA–DNA heteroduplex structure (called an R-loop) that extends from 200 bp upstream of the *COOLAIR* promoter for 300–700 nucleotides, sometimes reaching the *COOLAIR* proximal polyadenylation site. The R-loop suppresses *COOLAIR* transcription, probably through prevention of RNA polymerase II elongation (Sun *et al*., 2013). *COOLAIR* transcription through the R-loop is promoted by the P-TEFb transcription elongation complex (Wang *et al*., 2014). Although it was isolated specifically as a *COOLAIR* regulator, a mutation in the gene encoding AtNDX1 increased both *COOLAIR* and *FLC* expression in the endogenous gene context (Sun *et al*., 2013), demonstrating the tight link between sense and antisense transcription. Such sense/antisense coordination may be aided by the presence of an *FLC* gene loop, which involves physical interaction of the 5′ and 3′ regions (Crevillen *et al*., 2012), and/or the antisense transcription unit fully encompassing the sense transcription unit (Swiezewski *et al*., 2009). Another possibility is that sense or antisense transcription may influence the likelihood of a subsequent transcriptional event through modulation of the *FLC* chromatin environment.

## Conclusion

The many regulatory inputs make *FLC* appear a very complex locus. However, as our understanding progresses, a conceptually simple mechanism is emerging. We see *FLC* regulation as a chromatin state switching system. *FLC* chromatin has an ON state, is switched to an OFF state by the environment (over-wintering) or through genotype, and is then switched back to the ON state by reprogramming (reversing the switch). Both the maintenance of states and switching between states involves an intimate connection between chromatin regulators and sense/antisense RNA transcription and processing – a mechanism that has yet to be fully elaborated. Nuclear organization is an additional layer of regulation.

Cell-autonomous epigenetic switching performs the biological function of registering and remembering unpredictable and noisy temperature signals. An emerging theme is that the relative stabilities of activated and repressed epigenetic states appear to have been subtly modulated in natural accessions by cis sequence variation to generate a range of *FLC* haplotypes with characteristic responses to cold exposure. This may be because many of the regulators of *FLC* (such as Polycomb/Trithorax and the RNA 3′ processing machinery) are not specific to *FLC* regulation but instead perform more general tasks all over the genome. *FLC* is unlikely to be exceptional with respect to gene regulation; it has just been studied in more detail than most genes. Whenever an adaptive trait depends so closely on quantitative gene expression levels, subtle changes in regulation have strong consequences on fitness. In these cases, we may expect a similar level of complexity in gene regulation. As such, *FLC* continues to provide a valuable paradigm for studies of chromatin-based gene regulation, environmental perception and decision making.

## References

[b1] Adrian J, Farrona S, Reimer JJ, Albani MC, Coupland G, Turck F (2010). Cis-regulatory elements and chromatin state coordinately control temporal and spatial expression of FLOWERING LOCUS T in Arabidopsis. Plant Cell.

[b2] Aikawa S, Kobayashi MJ, Satake A, Shimizu KK, Kudoh H (2010). Robust control of the seasonal expression of the Arabidopsis FLC gene in a fluctuating environment. Proc. Natl Acad. Sci. USA.

[b3] Alló M, Buggiano V, Fededa JP (2009). Control of alternative splicing through siRNA-mediated transcriptional gene silencing. Nat. Struct. Mol. Biol.

[b4] Angel A, Song J, Dean C, Howard M (2011). A Polycomb-based switch underlying quantitative epigenetic memory. Nature.

[b5] Angel A, Song J, Yang H, Questa JI, Dean C, Howard M (2015). Vernalizing cold is registered digitally at FLC. Proc. Natl Acad. Sci. USA.

[b6] Annunziato AT (2005). Split decision: what happens to nucleosomes during DNA replication?. J. Biol. Chem.

[b7] Bastow R, Mylne JS, Lister C, Lippman Z, Martienssen RA, Dean C (2004). Vernalization requires epigenetic silencing of FLC by histone methylation. Nature.

[b8] Berr A, Xu L, Gao J, Cognat V, Steinmetz A, Dong A, Shen W-H (2009). SET DOMAIN GROUP25 encodes a histone methyltransferase and is involved in FLOWERING LOCUS C activation and repression of flowering. Plant Physiol.

[b500] Berry S, Hartley M, Olsson TSG, Dean C, Howard M (2015). Local chromatin environment of a Polycomb target gene instructs its own epigenetic inheritance. eLife.

[b9] Bonasio R, Tu S, Reinberg D (2010). Molecular signals of epigenetic states. Science.

[b10] Buzas DM, Robertson M, Finnegan EJ, Helliwell CA (2011). Transcription-dependence of histone H3 lysine 27 trimethylation at the Arabidopsis polycomb target gene FLC. Plant J.

[b11] Cao R, Wang L, Wang H, Xia L, Erdjument-Bromage H, Tempst P, Jones RS, Zhang Y (2002). Role of histone H3 lysine 27 methylation in Polycomb-group silencing. Science.

[b12] Castaings L, Bergonzi S, Albani MC, Kemi U, Savolainen O, Coupland G (2014). Evolutionary conservation of cold-induced antisense RNAs of FLOWERING LOCUS C in *Arabidopsis thaliana* perennial relatives. Nat. Commun.

[b13] Chan SWL, Henderson IR, Jacobsen SE (2005). Gardening the genome: DNA methylation in *Arabidopsis thaliana*. Nat. Rev. Genet.

[b14] Choi J, Hyun Y, Kang M-J (2009). Resetting and regulation of Flowering Locus C expression during Arabidopsis reproductive development. Plant J.

[b15] Coustham V, Li P, Strange A, Lister C, Song J, Dean C (2012). Quantitative modulation of polycomb silencing underlies natural variation in vernalization. Science.

[b16] Crevillen P, Dean C (2010). Regulation of the floral repressor gene FLC: the complexity of transcription in a chromatin context. Curr. Opin. Plant Biol.

[b17] Crevillen P, Sonmez C, Wu Z, Dean C (2012). A gene loop containing the floral repressor FLC is disrupted in the early phase of vernalization. EMBO J.

[b18] Crevillen P, Yang H, Cui X, Greeff C, Trick M, Qiu Q, Cao X, Dean C (2014). Epigenetic reprogramming that prevents transgenerational inheritance of the vernalized state. Nature.

[b19] Csorba T, Questa JI, Sun Q, Dean C (2014). Antisense COOLAIR mediates the coordinated switching of chromatin states at FLC during vernalization. Proc. Natl Acad. Sci. USA.

[b20] Davidovich C, Zheng L, Goodrich KJ, Cech TR (2013). Promiscuous RNA binding by Polycomb repressive complex 2. Nat. Struct. Mol. Biol.

[b21] De Lucia F, Crevillen P, Jones AME, Greb T, Dean C (2008). A PHD-polycomb repressive complex 2 triggers the epigenetic silencing of FLC during vernalization. Proc. Natl Acad. Sci. USA.

[b22] Deal RB, Henikoff JG, Henikoff S (2010). Genome-wide kinetics of nucleosome turnover determined by metabolic labeling of histones. Science.

[b23] Deng W, Buzas DM, Ying H, Robertson M, Taylor J, Peacock WJ, Dennis ES, Helliwell CA (2013). Arabidopsis Polycomb Repressive Complex 2 binding sites contain putative GAGA factor binding motifs within coding regions of genes. BMC Genom.

[b24] Derkacheva M, Steinbach Y, Wildhaber T, Mozgová I, Mahrez W, Nanni P, Bischof S, Gruissem W, Hennig L (2013). Arabidopsis MSI1 connects LHP1 to PRC2 complexes. EMBO J.

[b25] Dodd IB, Micheelsen MA, Sneppen K, Thon G (2007). Theoretical analysis of epigenetic cell memory by nucleosome modification. Cell.

[b26] Ferrell JE (2002). Self-perpetuating states in signal transduction: positive feedback, double-negative feedback and bistability. Curr. Opin. Cell Biol.

[b27] Ferrell JE (2012). Bistability, bifurcations, and Waddington’s epigenetic landscape. Curr. Biol.

[b28] Finnegan EJ, Kovac KA, Jaligot E, Sheldon CC, James Peacock W, Dennis ES (2005). The downregulation of FLOWERING LOCUS C (FLC) expression in plants with low levels of DNA methylation and by vernalization occurs by distinct mechanisms. Plant J.

[b29] Francis NJ, Follmer NE, Simon MD, Aghia G, Butler JD (2009). Polycomb proteins remain bound to chromatin and DNA during DNA replication in vitro. Cell.

[b30] Gan E-S, Xu Y, Wong J-Y, Goh JG, Sun B, Wee W-Y, Huang J, Ito T (2014). Jumonji demethylases moderate precocious flowering at elevated temperature via regulation of FLC in Arabidopsis. Nat. Commun.

[b31] Gaydos LJ, Wang W, Strome S (2014). H3K27me and PRC2 transmit a memory of repression across generations and during development. Science.

[b32] Gendall AR, Levy YY, Wilson A, Dean C (2001). The VERNALIZATION 2 gene mediates the epigenetic regulation of vernalization in Arabidopsis. Cell.

[b33] Greb T, Mylne JS, Crevillen P, Geraldo N, An H, Gendall AR, Dean C (2007). The PHD finger protein VRN5 functions in the epigenetic silencing of Arabidopsis FLC. Curr. Biol.

[b34] Hansen KH, Bracken AP, Pasini D, Dietrich N, Gehani SS, Monrad A, Rappsilber J, Lerdrup M, Helin K (2008). A model for transmission of the H3K27me3 epigenetic mark. Nat. Cell Biol.

[b35] Hazelbaker DZ, Marquardt S, Wlotzka W, Buratowski S (2013). Kinetic competition between RNA Polymerase II and Sen1-dependent transcription termination. Mol. Cell.

[b36] He Y, Doyle MR, Amasino RM (2004). PAF1-complex-mediated histone methylation of FLOWERING LOCUS C chromatin is required for the vernalization-responsive, winter-annual habit in Arabidopsis. Genes Dev.

[b37] He Y, Michaels SD, Amasino RM (2003). Regulation of flowering time by histone acetylation in Arabidopsis. Science.

[b38] Helliwell CA, Anderssen RS, Robertson M, Finnegan EJ (2015). How is FLC repression initiated by cold?. Trends Plant Sci.

[b39] Helliwell CA, Robertson M, Finnegan EJ, Buzas DM, Dennis ES (2011). Vernalization-repression of Arabidopsis FLC requires promoter sequences but not antisense transcripts. PLoS ONE.

[b40] Henikoff S, Shilatifard A (2011). Histone modification: cause or cog?. Trends Genet.

[b41] Heo JB, Sung S (2011). Vernalization-mediated epigenetic silencing by a long intronic noncoding RNA. Science.

[b42] Hepworth SR, Valverde F, Ravenscroft D, Mouradov A, Coupland G (2002). Antagonistic regulation of flowering-time gene SOC1 by CONSTANS and FLC via separate promoter motifs. EMBO J.

[b43] Herzog VA, Lempradl A, Trupke J (2014). A strand-specific switch in noncoding transcription switches the function of a Polycomb/Trithorax response element. Nat. Genet.

[b44] Holec S, Berger F (2012). Polycomb group complexes mediate developmental transitions in plants. Plant Physiol.

[b45] Hornyik C, Terzi LC, Simpson GG (2010). The spen family protein FPA controls alternative cleavage and polyadenylation of RNA. Dev. Cell.

[b46] Ietswaart R, Wu Z, Dean C (2012). Flowering time control: another window to the connection between antisense RNA and chromatin. Trends Genet.

[b47] Jamai A, Imoberdorf RM, Strubin M (2007). Continuous histone H2B and transcription-dependent histone H3 exchange in yeast cells outside of replication. Mol. Cell.

[b48] Jiang D, Gu X, He Y (2009). Establishment of the winter-annual growth habit via FRIGIDA-mediated histone methylation at FLOWERING LOCUS C in Arabidopsis. Plant Cell.

[b49] Johnson L, Mollah S, Garcia BA, Muratore TL, Shabanowitz J, Hunt DF, Jacobsen SE (2004). Mass spectrometry analysis of Arabidopsis histone H3 reveals distinct combinations of post-translational modifications. Nucleic Acids Res.

[b50] Kaneko S, Son J, Bonasio R, Shen SS, Reinberg D (2014). Nascent RNA interaction keeps PRC2 activity poised and in check. Genes Dev.

[b51] Kaneko S, Son J, Shen SS, Reinberg D, Bonasio R (2013). PRC2 binds active promoters and contacts nascent RNAs in embryonic stem cells. Nat. Struct. Mol. Biol.

[b52] Kaufman PD, Rando OJ (2010). Chromatin as a potential carrier of heritable information. Curr. Opin. Cell Biol.

[b53] Keller C, Kulasegaran-Shylini R, Shimada Y, Hotz H-R, Bühler M (2013). Noncoding RNAs prevent spreading of a repressive histone mark. Nat. Struct. Mol. Biol.

[b54] Kim SY, He Y, Jacob Y, Noh Y-S, Michaels S, Amasino RM (2005). Establishment of the vernalization-responsive, winter-annual habit in Arabidopsis requires a putative histone H3 methyl transferase. Plant Cell.

[b55] Klose RJ, Cooper S, Farcas AM, Blackledge NP, Brockdorff N (2013). Chromatin sampling – an emerging perspective on targeting polycomb repressor proteins. PLoS Genet.

[b56] Koornneef M, Alonso-Blanco C, Peeters AJM, Soppe W (1998). Genetic control of flowering time in Arabidopsis. Annu. Rev. Plant Physiol. Plant Mol. Biol.

[b57] Koornneef M, Hanhart CJ, van der Veen JH (1991). A genetic and physiological analysis of late flowering mutants in *Arabidopsis thaliana*. Mol. Gen. Genet.

[b58] Lee I, Michaels SD, Masshardt AS, Amasino RM (1994). The late-flowering phenotype of FRIGIDA and mutations in LUMINIDEPENDENS is suppressed in the Landsberg erecta strain of Arabidopsis. Plant J.

[b59] Lee J, Yun J-Y, Zhao W, Shen W-H, Amasino RM (2015). A methyltransferase required for proper timing of the vernalization response in Arabidopsis. Proc. Natl Acad. Sci. USA.

[b60] Li B, Carey M, Workman JL (2007). The role of chromatin during transcription. Cell.

[b61] Li D, Liu C, Shen L (2008). A repressor complex governs the integration of flowering signals in Arabidopsis. Dev. Cell.

[b62] Li P, Filiault D, Box MS (2014). Multiple FLC haplotypes defined by independent cis-regulatory variation underpin life history diversity in *Arabidopsis thaliana*. Genes Dev.

[b63] Li P, Tao Z, Dean C (2015). Phenotypic evolution through variation in splicing of the noncoding RNA COOLAIR. Genes Dev.

[b64] Liu FQ, Marquardt S, Lister C, Swiezewski S, Dean C (2010). Targeted 3’ processing of antisense transcripts triggers Arabidopsis FLC chromatin silencing. Science.

[b65] Liu FQ, Quesada V, Crevillen P, Bäurle I, Swiezewski S, Dean C (2007). The Arabidopsis RNA-binding protein FCA requires a lysine-specific demethylase 1 homolog to downregulate FLC. Mol. Cell.

[b66] Macknight R, Bancroft I, Page T (1997). FCA, a gene controlling flowering time in Arabidopsis, encodes a protein containing RNA-binding domains. Cell.

[b67] Margueron R, Reinberg D (2011). The Polycomb complex PRC2 and its mark in life. Nature.

[b68] Margueron R, Justin N, Ohno K (2009). Role of the polycomb protein EED in the propagation of repressive histone marks. Nature.

[b69] Marquardt S, Raitskin O, Wu Z, Liu FQ, Sun Q, Dean C (2014). Functional consequences of splicing of the antisense transcript COOLAIR on FLC transcription. Mol. Cell.

[b70] de la Mata M, Alonso CR, Kadener S, Fededa JP, Blaustein M, Pelisch F, Cramer P, Bentley D, Kornblihtt AR (2003). A slow RNA polymerase II affects alternative splicing in vivo. Mol. Cell.

[b71] Michaels SD, Amasino RM (1999). FLOWERING LOCUS C encodes a novel MADS domain protein that acts as a repressor of flowering. Plant Cell.

[b72] Moazed D (2011). Mechanisms for the inheritance of chromatin states. Cell.

[b73] Murray SC, Serra Barros A, Brown DA, Dudek P, Ayling J, Mellor J (2012). A pre-initiation complex at the 3’-end of genes drives antisense transcription independent of divergent sense transcription. Nucleic Acids Res.

[b74] Mylne JS, Barrett L, Tessadori F, Mesnage S, Johnson L, Bernatavichute YV, Jacobsen SE, Fransz P, Dean C (2006). LHP1, the Arabidopsis homologue of HETEROCHROMATIN PROTEIN1, is required for epigenetic silencing of FLC. Proc. Natl Acad. Sci. USA.

[b75] Oppenheim AB, Kobiler O, Stavans J, Court DL, Adhya S (2005). Switches in bacteriophage lambda development. Annu. Rev. Genet.

[b76] Pengelly AR, Copur O, Jackle H, Herzig A, Müller J (2013). A histone mutant reproduces the phenotype caused by loss of histone-modifying factor Polycomb. Science.

[b77] Petruk S, Sedkov Y, Johnston DM (2012). TrxG and PcG proteins but not methylated histones remain associated with DNA through replication. Cell.

[b78] Pien S, Fleury D, Mylne JS, Crevillen P, Inzé D, Avramova Z, Dean C, Grossniklaus U (2008). ARABIDOPSIS TRITHORAX1 dynamically regulates FLOWERING LOCUS C activation via histone 3 lysine 4 trimethylation. Plant Cell.

[b79] Ptashne M (2004). A Genetic Switch.

[b80] Ptashne M (2007). On the use of the word ‘epigenetic’. Curr. Biol.

[b81] Quesada V (2003). Autoregulation of FCA pre-mRNA processing controls Arabidopsis flowering time. EMBO J.

[b82] Riising EM, Comet I, Leblanc B, Wu X, Johansen JV, Helin K (2014). Gene silencing triggers polycomb repressive complex 2 recruitment to CpG islands genome wide. Mol. Cell.

[b83] Roeder AHK, Yanofsky MF (2006). Fruit development in Arabidopsis. Arabidopsis Book.

[b84] Rosa S, De Lucia F, Mylne JS, Zhu D, Ohmido N, Pendle A, Kato N, Shaw P, Dean C (2013). Physical clustering of FLC alleles during Polycomb-mediated epigenetic silencing in vernalization. Genes Dev.

[b85] Satake A, Iwasa Y (2012). A stochastic model of chromatin modification: cell population coding of winter memory in plants. J. Theor. Biol.

[b86] Schomburg FM, Patton DA, Meinke DW, Amasino RM (2001). FPA, a gene involved in floral induction in Arabidopsis, encodes a protein containing RNA-recognition motifs. Plant Cell.

[b87] Shafiq S, Berr A, Shen W-H (2014). Combinatorial functions of diverse histone methylations in *Arabidopsis thaliana* flowering time regulation. New Phytol.

[b88] Sheldon CC, Burn JE, Perez PP, Metzger J, Edwards JA, Peacock WJ, Dennis ES (1999). The FLF MADS box gene: a repressor of flowering in Arabidopsis regulated by vernalization and methylation. Plant Cell.

[b89] Sheldon CC, Conn AB, Dennis ES, Peacock WJ (2002). Different regulatory regions are required for the vernalization-induced repression of FLOWERING LOCUS C and for the epigenetic maintenance of repression. Plant Cell.

[b90] Sheldon CC, Hills MJ, Lister C, Dean C, Dennis ES, Peacock WJ (2008). Resetting of FLOWERING LOCUS C expression after epigenetic repression by vernalization. Proc. Natl Acad. Sci. USA.

[b91] Sheldon CC, Rouse DT, Finnegan EJ, Peacock WJ, Dennis ES (2000). The molecular basis of vernalization: the central role of FLOWERING LOCUS C (FLC). Proc. Natl Acad. Sci. USA.

[b92] Shindo C, Lister C, Crevillen P, Nordborg M, Dean C (2006). Variation in the epigenetic silencing of FLC contributes to natural variation in Arabidopsis vernalization response. Genes Dev.

[b93] Simpson GG, Dijkwel PP, Quesada V, Henderson IR, Dean C (2003). FY is an RNA 3′ end-processing factor that interacts with FCA to control the Arabidopsis floral transition. Cell.

[b94] Steffen PA, Ringrose L (2014). What are memories made of? How Polycomb and Trithorax proteins mediate epigenetic memory. Nat. Rev. Mol. Cell Biol.

[b95] Strange A, Li P, Lister C, Anderson J, Warthmann N, Shindo C, Irwin J, Nordborg M, Dean C (2011). Major-effect alleles at relatively few loci underlie distinct vernalization and flowering variation in Arabidopsis accessions. PLoS ONE.

[b96] Stuwe E, Tóth KF, Aravin AA (2014). Small but sturdy: small RNAs in cellular memory and epigenetics. Genes Dev.

[b97] Sun Q, Csorba T, Skourti-Stathaki K, Proudfoot NJ, Dean C (2013). R-loop stabilization represses antisense transcription at the Arabidopsis FLC locus. Science.

[b98] Sung S, Amasino RM (2004). Vernalization in *Arabidopsis thaliana* is mediated by the PHD finger protein VIN3. Nature.

[b99] Sung S, He Y, Eshoo TW, Tamada Y, Johnson L, Nakahigashi K, Goto K, Jacobsen SE, Amasino RM (2006a). Epigenetic maintenance of the vernalized state in *Arabidopsis thaliana* requires LIKE HETEROCHROMATIN PROTEIN 1. Nat. Genet.

[b100] Sung S, Schmitz RJ, Amasino RM (2006b). A PHD finger protein involved in both the vernalization and photoperiod pathways in Arabidopsis. Genes Dev.

[b101] Swiezewski S, Crevillen P, Liu FQ, Ecker JR, Jerzmanowski A, Dean C (2007). Small RNA-mediated chromatin silencing directed to the 3’ region of the Arabidopsis gene encoding the developmental regulator, FLC. Proc. Natl Acad. Sci. USA.

[b102] Swiezewski S, Liu FQ, Magusin A, Dean C (2009). Cold-induced silencing by long antisense transcripts of an Arabidopsis Polycomb target. Nature.

[b103] Takada S, Goto K (2003). Terminal flower2, an Arabidopsis homolog of heterochromatin protein1, counteracts the activation of flowering locus T by constans in the vascular tissues of leaves to regulate flowering time. Plant Cell.

[b104] Tamada Y, Yun J-Y, Woo SC, Amasino RM (2009). ARABIDOPSIS TRITHORAX-RELATED7 is required for methylation of lysine 4 of histone H3 and for transcriptional activation of FLOWERING LOCUS C. Plant Cell.

[b105] Tsai M-C, Manor O, Wan Y, Mosammaparast N, Wang JK, Lan F, Shi Y, Segal E, Chang HY (2010). Long noncoding RNA as modular scaffold of histone modification complexes. Science.

[b106] Turck F, Coupland G (2014). Natural variation in epigenetic gene regulation and its effects on plant developmental traits. Evolution.

[b107] Turck F, Roudier F, Farrona S (2007). Arabidopsis TFL2/LHP1 specifically associates with genes marked by trimethylation of histone H3 lysine 27. PLoS Genet.

[b108] Veening J-W, Smits WK, Kuipers OP (2008). Bistability, epigenetics, and bet-hedging in bacteria. Annu. Rev. Microbiol.

[b109] Vilar JMG, Guet CC, Leibler S (2003). Modeling network dynamics: the lac operon, a case study. J. Cell Biol.

[b110] Voigt P, LeRoy G, Drury WJ, Zee BM, Son J, Beck DB, Young NL, Garcia BA, Reinberg D (2012). Asymmetrically modified nucleosomes. Cell.

[b111] Wang R, Farrona S, Vincent C, Joecker A, Schoof H, Turck F, Alonso-Blanco C, Coupland G, Albani MC (2009). PEP1 regulates perennial flowering in *Arabis alpina*. Nature.

[b112] Wang ZW, Wu Z, Raitskin O, Sun Q, Dean C (2014). Antisense-mediated FLC transcriptional repression requires the P-TEFb transcription elongation factor. Proc. Natl Acad. Sci. USA.

[b113] Wigge PA (2011). FT, a mobile developmental signal in plants. Curr. Biol.

[b114] Wood CC, Robertson M, Tanner G, Peacock WJ, Dennis ES, Helliwell CA (2006). The *Arabidopsis thaliana* vernalization response requires a polycomb-like protein complex that also includes VERNALIZATION INSENSITIVE 3. Proc. Natl Acad. Sci. USA.

[b115] Yang H, Howard M, Dean C (2014). Antagonistic roles for H3K36me3 and H3K27me3 in the cold-induced epigenetic switch at Arabidopsis FLC. Curr. Biol.

[b116] Yuan W, Xu M, Huang C, Liu N, Chen S, Zhu B (2011). H3K36 methylation antagonizes PRC2-mediated H3K27 methylation. J. Biol. Chem.

[b117] Zhang X, Clarenz O, Cokus S, Bernatavichute YV, Pellegrini M, Goodrich J, Jacobsen SE (2007a). Whole-genome analysis of histone H3 lysine 27 trimethylation in Arabidopsis. PLoS Biol.

[b118] Zhang X, Germann S, Blus BJ, Khorasanizadeh S, Gaudin V, Jacobsen SE (2007b). The Arabidopsis LHP1 protein colocalizes with histone H3 Lys27 trimethylation. Nat. Struct. Mol. Biol.

[b119] Zhao Z, Yu Y, Meyer D, Wu C, Shen W-H (2005). Prevention of early flowering by expression of FLOWERING LOCUS C requires methylation of histone H3 K36. Nat. Cell Biol.

